# Is Greenfield investment improving welfare: A quantitative analysis for Latin American and Caribbean developing countries

**DOI:** 10.1016/j.heliyon.2023.e20703

**Published:** 2023-10-10

**Authors:** Ali Raza, Muhammad Imran Nadeem, Kanwal Ahmed, Izaz Hassan, Sayed M. Eldin, Nivin A. Ghamry

**Affiliations:** a Department of Management Sciences, The University of Haripur, Pakistan; b School of Computer and Artificial Intelligence, Zhengzhou University, Zhengzhou, 450001, China; c Department of Software Engineering, University of Science and Technology, Bannu, KP, Pakistan; d Center of Research, Faculty of Engineering, Future University in Egypt, New Cairo, 11835, Egypt; e Cairo University, Faculty of Computers and Artificial Intelligence, Giza, Egypt

**Keywords:** Greenfield investment, Foreign aid, Sys-GMM technique, Latin American and Caribbean developing countries

## Abstract

Greenfield investment is considered the backbone of emerging economies and developing countries. This research is carried out to investigate the causal impact of Greenfield investment as a target variable and some other controlled variables for the sample of 23 Latin American and Caribbean (LA&C) developing countries. The period is 1998–2017, and Levin, Lin and Chu (LLC) and System-Generalized Method of Moment (Sys-GMM) techniques are employed for analytical analysis. The Sys-GMM technique estimates show that Greenfield investment has a significant positive impact on these countries’ economic growth, health, education, and welfare. Furthermore, controlled variables remittances have a significant and positive impact, while foreign aid has a negative effect on the dependent variables. The rest of the other controlled variables show mixed results. From the analysis, it is suggested that Greenfield investment has improved per capita income, education and health sector that further enhanced the welfare of the society. In addition, new foreign investment creates job employment and brings innovations that improve labour skills. On the other hand, foreign aid must be avoided, which harms the economic activities of developing countries. Therefore, it is concluded that governments of Latin American and Caribbean developing countries adopt more friendly policies to attract Greenfield investment.

## 1 Introduction

Investment in a developing country is a blessing that generates employment, bring innovations and transfer new technologies that further improve labour skill. Many developing countries have improved their economic activities through foreign investments. Globalisation, especially Foreign Direct Investment (FDI), is the most efficient investment that contributes to economic growth [1]. FDI is considered best suitable for developing countries because these countries have less capital but an abundance of natural resources [2]. The importance of FDI can be seen in developing countries that improve education and labour skills by transferring the latest technology from developed countries [3]. The flow of FDI to the globe declined to 1.3 trillion US$ from 1.5 trillion US$, but to developing countries, there was a 2 % rise in the year 2018 [4]. The FDI inflow to developing economies is around 685 billion US$ for the year 2019 [5].

Nations all throughout the globe, particularly those with insufficient assets, fights intensely for FDI, with re-searcher examining the many factors that influence FDI [6]. FDI can be divided into different modes as Greenfield- FDI (GFDI), Brownfield-FDI and Merger & Acquisition (M&A) [7]. Greenfield investment is the new startup in a host developing country by creating new jobs for the locals that further brings competition in the market. Investors from developed countries invest in the form of Greenfield rather than M&A [8]. With Greenfield investment as new and latest, technological productions start that increases the efficiency of firms [9]. Greenfield investment is thought to have a favourable impact on the economy of the host country since it can raise capital stock, create jobs, and accelerate growth [10]. According to the study of [11], Greenfield investment has improved health due to increase in per capita income. Greenfield brings job to the host country, creating awareness for the best health facilities, access to the modern education and improved the socioeconomic status of poor nations. Greenfield investment is more suitable for developing countries as compared to M&A [12]. Because in these nations a very low price for fixing new plant setup exists. Greenfield investment earns a very low profit in those countries which are less attractive or there exists strict rules of FDI policy.

Recently Greenfield investment to the developed countries dropped to 132 billion US$, but for developing countries, it rises to 582 billion US$ for the year 2018 [4]. Greenfield investments spur economic growth of developing countries with the compromise on the pollution haven hypothesis [13]. On the other hand, as compared to other forms of FDI, Greenfield investment showed high profit for the developed countries and developing countries [14]. Green-field investment is welcomed in developing countries by following no strict environmental protocol. With the increase in such investment, there is a supportive image on the health and education of the African developing countries that improve welfare and increase per capita income [15]. The objectives of the study are.

1. To explore the effect of Greenfield investment on socioeconomic development in LA&C developing countries.
2. To investigate the effect of Greenfield investment on economic growth in LA&C developing countries.
3. To analyze the effect of Greenfield investment on health in LA&C developing countries.
4. To assess the effect of Greenfield investment on education in LA&C developing countries.

Earlier studies about FDI inflows were mostly driven by a “market-seeking” motive. This suggests that the foreign investors were interested to make access to a host country domestic market, which offered prospective prospects for more sales and profits. But as time went on, the “efficiency-seeking” motive took center stage. By utilising certain factors of the host nation, such as cheaper manufacturing costs, skilled labor, advantageous regulations, or cutting-edge technology, international investors can increase their efficiency and productivity. Similarly, majority of developing countries face a capital deficit, which is represented in their respective “savings investment” and “import export” gaps. This suggests that developing countries lack the foreign currency necessary to finance their investment requirements. So foreign capital inflows especially Greenfield investment, remittances and foreign assistance will bridge up this gap of developing countries. Till late 1970's LA&C countries maintained strict foreign investment regulations but later on these countries shifted their attitude towards welcoming foreign investment policies. Foreign investment was welcomed all over the world, both in developed and developing countries. There was a declined tendency in other forms of capital inflows showing how these forms of capital were discouraged.

This study is mainly related to the FDI mode of Greenfield investment and the benefits of this mode in the host LA&C countries. Other studies mainly discussed the importance of Greenfield investment versus economic growth of developed and developing countries. Literature also shows that some studies are very related to either theoretical based or empirical based without related theories but this study is different form rest of literature in terms of theoretical based as well as empirical based study. This study is based on the theories related to economic growth, health, education and welfare for each of the dependent variable.

LA&C developing nations has improved institutional, governance, and anti-corruption frameworks supporting business confidence. Economic growth boosted by investment, productivity growth, attractive business climate and the high quality of infrastructure and education [16]. The LA&C nations shared almost 12 % to total Greenfield investment and this share would further increase to 13.6 % in 2023 [5]. Most of the LA&C nations has improved their per capita income and move to upper middle-income countries. This study is conducted due to three main reasons. First, in literature, there is a gap to find the association between Greenfield investment and LA&C developing nation's economic progression, and this study fills this gap. Second, no such research has been conducted so far to find the impact of Greenfield investment on health, education and the overall welfare, and this study will fill this gap. Third, specific Greenfield investment data is used to carry out this research in the literature. The literature studies used Greenfield-FDI data as Greenfield projects that mislead the analysis. Greenfield investment can be calculated in terms of the difference between total FDI inflow and M&A sales [2,7]. Before 2003 there was no actual data of Greenfield investment but rather to use Greenfield projects data. In the year 2004, research was done by introducing this rule by Ref. [17], later on, followed by many researchers in the literature.

### 1.1 Sampled LA&C countries are

Argentina, Belize, Bolivia, Brazil, Chile, Colombia, Costa Rica, Cuba, Dominican Republic, El Salvador, Ecuador, Guatemala, Guyana, Haiti, Honduras, Jamaica, panama, Mexico, Nicaragua, Paraguay, Peru, Trinidad and Tobago, Venezuela.

## 2 Literature review

Literature is full of studies conducted on the applied and secondary data both for developing and developed countries representing mathematical equations, but few studies are also conducted on the basis of empirical work representing in-depth discussion on various aspects related to different issues [12]. considered a partial equilibrium model both for host and foreign countries with factors of cost difference, market size, market competition and fixed cost. These four factors were associated with Greenfield investment and M&A. The study concluded on the basis of these factors that M&A is more attractive and beneficial for production which is also supported by the mentioned model. Similarly other studies discuss either production productivity curve, FDI stock, portfolio investment or joint venture. This current study focuses on the health, education, economic growth and socioeconomic aspect of LA&C countries. This study has mostly discussed those studies that worked on applied and secondary data.

Growth theoretic models have been used as a framework for analysing the growth-enhancing benefits of Greenfield- FDI at the macro level. Based on the “advantage of backwardness,” the Solow-Swan model [18,19] predicts that the deeper a country is behind the global technology frontier, the faster it will grow. This relative backwardness, in turn, provides emerging countries with an incentive to save and invest, resulting in a narrowing of the gap between them and their developed counterparts. Due to declining returns to capital, the typical neoclassical model implies that.

Greenfield-FDI will only affect growth in the short run, but will have no effect on long-run growth [20]. The rise of endogenous growth models has given rise to a new way of thinking about the FDI-growth link. These models claim that while relative backwardness in a developing country has the potential for rapid growth, the amount to which this promise is achieved is determined by the domestic economy's ability to absorb and adapt foreign technologies to local conditions [21]. That is, productivity and income disparities between countries tend to shrink over time, but only un-der certain country-specific circumstances. Endogenous growth models provide a supportive framework for assessing the FDI-development link if FDI is considered as an accelerator for productivity growth, domestic investment, and technical progress since they allow for long-run growth due to externalities.

[22] conducted one of the pioneer studies and established a positive connection between Brownfield-FDI and economic growth of Chinese, Hong Kong and South Korean economies. The author uses the diamond model and supports that the Brownfield-FDI has stimulated economic growth in those countries from 1999 to 2002 [17]. findings confirmed that both Brownfield-FDI and Greenfield-FDI have favourable impact on economic improvement. For a sample of 72 nations from 1978 to 2003, the research used a vector self-regression estimator. For 53 nations sample,

[23] employed a Random Effect Model, from 1996 to 2006. Granger causality tests have shown that Greenfield-FDI is one way to cause economic growth, M&A, on the other hand, has negative impact on developed nation's economic improvement. This study concluded that Greenfield-FDI brings innovation and new technology to the host county as the advanced countries firms transfer capital for investment.

[24] studied investment companies from rich nations, for a sample of 35 developing nations and justified their preference for investment in culturally distant lands through Greenfield-FDI. While [10] stated that Greenfield-FDI contributes to accelerating economic growth, M&A does only contribute to an increase in capital levels. In addition, the study used instrumental experiments on a sample of 84 nations from 1987 to 2001 and assessed that Greenfield- FDI had induced both the developing and developed economies in economic development [25]. applied a bound test method to operate on Malaysia's time-series data from 1970 to 2009. The study explored the fact that Greenfield-FDI stimulated economic growth and strengthened economic growth in Malaysia. Similarly [12] supported that M&A is more supportive than Greenfield investment in maximizing the domestic welfare depending on the economic condition of the firm. The study also recommends a policy implication for the best suitable choice of host firm for foreign firms for merger if the investors choose cross border merger.

To analyze the sample of 40 developing countries from 1990 to 2009 [26], used a Sys-GMM estimator. The study showed that GDP is more narrowly linked to M&A per capita than to Greenfield-FDI. Between 1990 and 2009, Greenfield-FDI and Brownfield-FDI inflows in 93 countries contrasted with [27]. The study found the effect of Greenfield-FDI on income inequality was significantly positive, while the influence of Brownfield-FDI on income disparity was not significant.

[28] researched 78 sampled developing countries from 1978 to 2005 and used GMM estimators. The study's results indicated the important link between M&A and Greenfield-FDI to economic progress; moreover, the influence of the Greenfield-FDI on economic growth is greater than the impact of M&A. The authors concluded that host countries receive capital and the latest technology from developing countries that helps strengthen economic activities and generate employment. In the [29] analysis for the sample of the MENA countries, similar findings were also found. Therefore, the study established that M&A does not lead to the capital expansion of the host nation.

Working on a sample of 135 countries [30], used the GMM estimation technique for 2003–2012. The research established that Greenfield-FDI had positive repercussions on trade in the total sampled nations, while M&A played no part [31]. establish a negative impact of Greenfield-FDI and Brownfield-FDI on economic progress in 12 European Union nations for the year 1999–2010 [8]. found that Greenfield-FDI, the companies that invest in Greenfield-FDI, will lower the efficiency and welfare of the host nation.

[32] also used GMM estimators on data from 2003 to 2014. The author believed Greenfield-FDI and M&A had contributed positively to stimulating economic development, and developing nations would get advantage more if the levels of human capital were increased [33]. highlighted the analysis of Greenfield-FDI and M&A used by transactional corporations. The study concluded that transactional corporation's preferred GFDI in such a situation while supporting long-term growth [34]. matched 123 sampled nations, including developing and developed nations, from 2003 to 2011. The study revealed that Greenfield-FDI did not contribute to the overall productivity factor. Furthermore, in developing countries, both Greenfield-FDI and M&A had no significant impact on productivity.

[7] analyzed the influence of M&A and Greenfield-FDI on the economic progress of European Union sampled nations for the year 2003–2015. Research shows that through the Basher and Westerlund co-integration test, M&A and Greenfield-FDI have beneficial effects on economic growth. To evaluate the association between inward Greenfield- FDI and labour polarization [35], examined European Union sampled countries. The study confirmed that Greenfield- FDI helps in the progress of low skill occupation. Similarly [36] investigated the European Union nations sample and reported that Greenfield-FDI boosts host country investment.

[37] revealed that Greenfield investment is the best possible option for politicians to announce new projects for the attraction of voters. The study assessed that Greenfield investment is consider as tool that increases the chances of re-election with no influence on the results decisions [38]. employed the GMM method on 131 sampled countries from 2003 to 2015. Corruption has a significant positive effect on Greenfield-FDI in developing nations, while it has a major negative influence in developed countries. Work ing on the sampled countries of ASEAN and SAARC [13], revealed that M&A had compromised the atmosphere and Greenfield-FDI using random effect and Robust Least Square method for 2003 to 2014.

[15] worked on the time series data of Pakistan from 1990 to 2018 using the ARDL technique and assessed that Greenfield-FDI is advantageous for health improvement condition and boosting individual income of a host nation society. The study also confirmed that Greenfield-FDI also helps in the welfare of a country. Similarly [2] high-lighted the case of African developing countries from 1998 to 2017 using the GMM technique. The study showed that Greenfield-FDI improved the economic progress and economic development of African developing nations. The re-search also analyzed that Greenfield investment brings development in the individual economy and health conditions [39,40]. considered that air pollution as a cause for degrading the individual health. Similarly the increase in per capita income does not contribute to improve the health because of more pollution. These studies also revealed that the increase in pollution also affect life expectancy and cause more damages to health. The latest study of [41] revealed that MENA sampled developing countries have improved the living standard by getting education and health facilities due to the inflow of Greenfield investment to these countries. The research covers sampled MENA developing nations using the system GMM technique. It concludes that by adopting friendly business policies for foreign investors, the host country attracts maximum investment, improving labour health and their children's education.

## 3 Data

The LA&C nations are considered as the most attractive and favourable region for investment. LA&C nations have increased and contributed almost 26 % to the total global GDP share for year 2019. Most of the LA&C nations are middle income countries and their growth rate is about 6.5 % [42]. Variables used in this study as dependent and independent, and some controlled variables. The proxies and unit used for each variable is as, Greenfield investment is the differences between total FDI inflows and M&A in US$. Remittances is the amount of money send by migrants and foreign aid is the official development assistance in US$. The proxy for trade openness is the number of exports plus imports in US$, while population is the total number of citizen of a country. Current percentage of inflation for a current year is considered as the inflation of a specific country. The list of LA&C developing nations is taken from World Bank, which divided countries on the base of income per capita. This study chooses a sample of 23 LA&C developing countries and the period is from 1998 to 2017. The time interval is selected because of the accessibility of data. This period has observed a huge arrival of Greenfield investment to the LA&C developing nations and a quick progression of globalisation. The data is usually taken from the [5,42,43].

## 4 Models and technique

To accomplish the list of goals of this study, we use the following Generalized Method of Moment (GMM) used by prior studies and can be written as;

(1) $$Y_{\text{it}}=\alpha_{0}+\alpha_{1}Y_{\text{it}-1}+\alpha_{2}X_{\text{it}}+\alpha_{3}Z_{\text{it}}+\mu_{\text{it}}$$

where is the economic growth of each nation in time and is target variable and is set of controlled variables, while fluctuates through nations and time. The above equation (1) is the general form of the GMM model, and the rest of the models are based on this equation (1). This study utilized the Harrod-Domar theory as the authors first introduced investment into the economic growth model and later on used by Refs. [7,44] studies. Reordering equation (1)

(2) $$EG_{\text{it}}=\alpha_{0}+\alpha_{1}EG_{\text{it}-1}+\alpha_{2}\text{GFD}I_{\text{it}}+\alpha_{3}\text{RE}M_{\text{it}}+\alpha_{4}\text{OD}A_{\text{it}}+\alpha_{5}TO_{\text{it}}+\alpha_{6}\text{PO}P_{\text{it}}+\alpha_{7}\text{IN}F_{\text{it}}+\vartheta_{\text{it}}$$

### 4.1 GMM model for economic growth

Equation (3) is the final model for economic growth and is used in Refs. [7,44] studies.

(3) $$\begin{array}{l} EG_{it}=\alpha_{0}+\alpha_{1}E{G_{i}}_{\left(t-1\right)}+\sum_{j=0}^{p}\alpha_{2}GFD{{{I_{i}}_{(t-}}_{j}}_{)}+\sum_{j=0}^{q}\alpha_{3}RE{{{M_{i}}_{(t-}}_{j}}_{)}+\sum_{j=0}^{r}\alpha_{4}OD{{{A_{i}}_{(t-}}_{j}}_{)}+\\ \sum_{j=0}^{h}\alpha_{5}TO_{i\left(t-j\right)}+\sum_{j=0}^{s}\alpha_{6}POP_{i\left(t-j\right)}+\sum_{j=0}^{m}\alpha_{7}INF_{i\left(t-j\right)}+\vartheta_{it} \end{array}$$

Economic growth is the proxy used for income taken as the dependent variable while GFDI is the Greenfield investment as the target independent variable. The rest of the independent variables are controlled variables. This model is based on the Harrod-Domar theory as these economists add investment for the first time in classical economic growth equation. GFDI will add more investment opportunities to the employment of local skilled labor and with the transfer of new technologies to host countries will bring more efficiency to the productivity.

### 4.2 GMM model for health

Equation (4) is the final model for health. The health index model for health is based on [45] study and used in Ref. [40] study.

(4) $$\begin{array}{l} HEALTH_{it}=\alpha_{0}+\alpha_{1}HEALT{H_{i}}_{\left(t-1\right)}+\sum_{j=0}^{p}\alpha_{2}GFD{{{I_{i}}_{(t-}}_{j}}_{)}+\sum_{j=0}^{q}\alpha_{3}RE{{{M_{i}}_{(t-}}_{j}}_{)}+\sum_{j=0}^{r}\alpha_{4}OD{{{A_{i}}_{(t-}}_{j}}_{)}+\\ \sum_{j=0}^{h}\alpha_{5}TO_{i\left(t-j\right)}+\sum_{j=0}^{s}\alpha_{6}POP_{i\left(t-j\right)}+\sum_{j=0}^{m}\alpha_{7}INF_{i\left(t-j\right)}+\vartheta_{it} \end{array}$$

Health is the proxy used for life expectancy taken as dependent variable while GFDI is the Greenfield investment as target independent variable. Rest of the independent variables are controlled variables. The inclusion of health variable is related to human capital and this variable has great impact on the productivity in an economy. Higher expectancy rate will help in learning more technological skill will help to the efficiency of productivity. Skilled labor can be trained to efficiently utilize the new technology of the host country [20].

### 4.3 GMM model for education

Equation (5) is the final model for education. The model for the education index is used in Refs. [41,44] studies.

(5) $$\begin{array}{l} EDU_{it}=\alpha_{0}+\alpha_{1}ED{U_{i}}_{\left(t-1\right)}+\sum_{j=0}^{p}\alpha_{2}GFD{{{I_{i}}_{(t-}}_{j}}_{)}+\sum_{j=0}^{q}\alpha_{3}RE{{{M_{i}}_{(t-}}_{j}}_{)}+\sum_{j=0}^{r}\alpha_{4}OD{{{A_{i}}_{(t-}}_{j}}_{)}+\\ \sum_{j=0}^{h}\alpha_{5}TO_{i\left(t-j\right)}+\sum_{j=0}^{s}\alpha_{6}POP_{i\left(t-j\right)}+\sum_{j=0}^{m}\alpha_{7}INF_{i\left(t-j\right)}+\vartheta_{it} \end{array}$$

Education is the proxy used for schooling taken as dependent variable while GFDI is the Greenfield investment as target independent variable. Rest of the independent variables are controlled variables. This model is based on the endogenous growth theory developed by Ref. [46]. This model is of the view that long-term economic growth is largely influenced by investments in knowledge and education. More investment in terms of training and workshops will improve their skills that will further improve the quality of products.

### 4.4 GMM model for human development index

Equation (6) is the final model for development. The model for HDI is based on Human Development Theory (HDI) [47], which is also used by Refs. [44,48,49] in their research work.

(6) $$\begin{array}{l} HDI_{it}=\alpha_{0}+\alpha_{1}HD{I_{i}}_{\left(t-1\right)}+\sum_{j=0}^{p}\alpha_{2}GFD{{{I_{i}}_{(t-}}_{j}}_{)}+\sum_{j=0}^{q}\alpha_{3}RE{{{M_{i}}_{(t-}}_{j}}_{)}+\sum_{j=0}^{r}\alpha_{4}OD{{{A_{i}}_{(t-}}_{j}}_{)}+\\ \sum_{j=0}^{h}\alpha_{5}TO_{i\left(t-j\right)}+\sum_{j=0}^{s}\alpha_{6}POP_{i\left(t-j\right)}+\sum_{j=0}^{m}\alpha_{7}INF_{i\left(t-j\right)}+\vartheta_{it} \end{array}$$

The Human Development index is the proxy used for welfare or socioeconomic development taken as dependent variable while GFDI is the Greenfield investment as target independent variable. Rest of the independent variables are controlled variables. This model is based the human development index probably human capital theory developed b by Mahboob ul Haq in 1990 with the group of other economists. This theory mainly focuses on the productivity and efficiency increasing education, per capita income, skills, healthy environment and overall welfare of a society. Investment in human knowledge and health of people will not only improve their individual capacity of learning but will also improve the capability of critical thinking.

As pointed by Ref. [50], endogeneity occurs when a regressor is associated with the residual [51]. introduce a moment estimation method for panel data to control orthogonality and endogeneity but later on [52] establish a technique IV-estimator overcomes this problem [53]. then work on the new System GMM, which controls endogeneity by introducing lagged dependent instrumental variables that give accurate results. To ensure the model estimation is unbiased, this study uses an econometric technique which is more efficient in the presences of endogenous regressors. In panel analysis, Two Stage Least Squares is commonly employed to deal with endogeneity but GMM is superior and more efficient [48]. Precisely, system-GMM estimators assume endogeneity and use moment conditions to produce a set of effective instruments for endogenous regressors that can enhance efficiency greatly [48,53,54]. The validity of additional instruments, as well as the absence of second-order autocorrelation, are both required for Sys-GMM to work.

[51,52] provide the Sargan/Hansen test of over-identification and the Arellano-Bond (AR2) autocorrelation to examine these two circumstances. The over-identification Sargan/Hansen test, which verifies the instruments' valid-ity, and Arellano-Bond (AR2) autocorrelation, which verifies the lack of second-order autocorrelation. This study model's validity is ensured by the high p-values of these tests in robust estimations. Mover over the GMM estimator improves substantially the estimate of the impact of Greenfield investment on education, health, economic growth and overall welfare relative to the models which focus on within-country changes in these areas, adding information on cross-country variation. This study follows the System GMM technique for the analysis of the final results.

## 5 Results and discussion

Latin American and Caribbean region consists of mostly developing countries. This study's sub-sample of Latin American and Caribbean developing countries depends on the larger scale of inflows of Greenfield-FDI and remittances. Table 1, displays some of the important summary statistics of LA&C developing countries of all variables. Here, each panel dimension has 460 observations. The results of this region are given below.

**Table 1:** Descriptive statistic of LA&C developing countries.

Variables	Obs	Mean	Std. Dev.	Min	Max	Skewness	Kurtosis
GFDI_*it*_	460	2.6187	0.9497	−0.3888	4.9087	4.6756	29.0461
REM_*it*_	460	8.6767	0.8594	5.9531	10.4857	−0.4959	2.8743
ODA_*it*_	460	29.0816	67.8982	26.9004	555.8688	4.1275	23.8356
TO_*it*_	460	−0.1453	0.1904	−0.7841	0.3154	−0.3904	3.1463
POP_*it*_	460	6.6903	0.8078	5.2378	8.3207	−0.0129	2.3537
INF_*it*_	460	8.0895	16.7249	−7.1137	254.9485	9.5976	121.9272
EDU_*it*_	460	−0.2319	0.0786	0.4776	−0.1053	−1.2023	4.0099
HEALTH_*it*_	460	−0.1049	0.0382	−0.2426	−0.0343	−1.0965	4.4538
EG_*it*_	460	−0.2057	0.0710	−0.4168	−0.1048	−1.1274	3.7359
HDI_*it*_	460	−0.1808	0.0549	−0.3615	−0.1008	−1.3685	4.5973

Table 2 shows the results of the correlation matrix of all variables. It can be seen that Greenfield investment has a positive correlation with all variables except trade openness. Theoretically, Greenfield investment and trade openness have an indirect relationship [7]. Similarly, remittance has a positive correlation with all variables except inflation and trade. It is proved theoretically that remittances and trade are inversely related. When a country has high trade with other countries, labour without going abroad to earn livelihood [55]. ODA also negatively correlates with inflation and trade while a positive correlation with the rest of the variables. Trade has only a positive correlation with education while a negative correlation with other variables. When trade is made between countries, people get more education because of the exchange of products and goods [56]. A negative correlation exists between population and education, while a positive correlation with other variables is observed. Inflation has a negative correlation with education, health and HDI while a positive correlation with economic growth and this is proved theoretically by Ref. [57]. Further, HDI, economic growth, health and education show a positive correlation with each other.

**Table 2:** Correlation matrix of LA&C developing countries variables.

Variables	GFDI_*it*_	REM_*it*_	ODA_*it*_	TO_*it*_	POP_*it*_	INF_*it*_	EDU_*it*_	HEALTH_*it*_	EG_*it*_	HDI_*it*_
GFDI_*it*_	1									
REM_*it*_	0.703	1								
ODA_*it*_	0.673	0.423	1							
TO_*it*_	−0.593	−0.462	−0.567	1						
POP_*it*_	0.829	0758	0.625	−0.772	1					
INF_*it*_	0.046	−0.026	−0.034	−0.099	0.156	1				
EDU_*it*_	0.296	0.171	0.124	0.094	−0.010	−0.032	1			
HEALTH_*it*_	0.501	0.359	0.236	−0.175	0.241	−0.012	0.529	1		
EG_*it*_	0.683	0.489	0.381	−0.338	0.467	0.030	0.656	0.729	1	
HDI_*it*_	0.552	0.376	0.278	−0.142	0.253	−0.005	0.881	0.801	0.913	1

[58] introduce the cross-sectional dependence test among the panel data for the consistent and unbiased results. This study applied different cross-sectional tests for the conformation of influence of one cross-section over another cross-section. Table 3, is about cross-sectional dependence and the null hypothesis of no cross-sectional dependence is accepted due to high p-value. This study outcome indicates that the LA&C countries did not affect one another and so the shock of one country has no influence on the other country. In panel data analysis, the cross dependence test is used to identify and take into account the interdependence between individual observations within a panel dataset, enhancing the effectiveness and validity of statistical inferences, directing model selection, and providing policy implications.

**Table 3:** Cross-sectional Dependence tests.

Variables	Breusch-Pagan LM	Pesaran Scaled LM	Pesaran CD
GFDI_*it*_	43.646∗∗ (0.231)	23.239∗∗ (0.842)	8.982∗∗ (0.771)
REM_*it*_	34.591∗∗ (0.245)	18.423∗∗ (0.622)	6.855∗∗ (0.562)
ODA_*it*_	33.125∗∗ (0.311)	23.987∗∗ (0.556)	6.013∗∗ (0.341)
TO_*it*_	75.991∗∗ (0.298)	3.868∗∗ (0.479)	9.163∗∗ (0.228)
POP_*it*_	80.213∗∗ (0.376)	19.538∗∗ (0.557)	4.267∗∗ (0.375)
INF_*it*_	36.091∗∗ (0.344)	6.725∗∗ (0.775)	3.136∗∗ (0.598)
EDU_*it*_	23.626∗∗ (0.401)	13.193∗∗ (0.663)	3.295∗∗ (0.387)
HEALTH_*it*_	25.663∗∗ (0.443)	12.892∗∗ (0.705)	4.112∗∗ (0.658)
EG_*it*_	29.095∗∗ (0.264)	6.993∗∗ (0.499)	3.092∗∗ (0.226)
HDI_*it*_	26.458∗∗ (0.193)	74.774∗∗ (0.472)	13.995∗∗ (0.332)

Brackets (.) denotes *P*-Value, ** denotes 5 % significance level.

In panel data, heterogeneity is a serious issue, and most studies fail to capture heterogeneity in their data analysis for unit root testing. Later on [59], (LLC) solved this issue, and this study used the IPS method in data analysis for unit root testing. This study used Table 4, which indicates the outcomes of the panel unit-root in the presence of deterministic terms for all variables. It is observed that trade, inflation, ODA and Greenfield investment are identified as stationary at level (Null Hypothesis is rejected) if only constant or both constant and the trend is considered, thus suggesting a long term impact on the economic growth. However, population, remittances, HDI, economic growth and health are stationary either when only constant or both trend terms are included. Only education is identified as unit root at level (Null Hypothesis is accepted). But, at the first difference, the unit-root variables at the level now are shown to be stationary.

**Table 4:** Unit root tests of all variables of LA&C developing countries.

Variables	At Level		First Difference	
	Constant	Constant + Trend	Constant	Constant + Trend
GFDI_*it*_	−2.3248** (0.001)	−5.6502** (0.000)	–	–
REM_*it*_	−3.803** (0.001)	−0.124 (0.450)	–	−9.435** (0.000)
ODA_*it*_	−4.6938** (0.000)	−6.9196** (0.000)	–	–
TO_*it*_	−2.3453** (0.009)	−4.3319** (0.000)	–	–
POP_*it*_	−7.9669** (0.000)	4.9857 (0.995)	–	−3.8056** (0.001)
INF_*it*_	−6.9816** (0.000)	−8.1732** (0.000)	–	–
EDU_*it*_	−4.0289** (0.000)	2.6808 (0.996)	–	−2.5192** (0.005)
HEALTH_*it*_	0.845 (0.801)	−0.133 (0.447)	−8.7449** (0.000)	−9.9231** (0.000)
EG_*it*_	4.1193 (0.996)	−2.7047** (0.003)	−8.6823** (0.000)	–
HDI_*it*_	3.1826 (0.998)	−1.9431** (0.026)	−9.2465** (0.000)	–

Brackets (.) denote *P*-value, ** denotes 5 % significance level.

Table 5 indicates that the constant term is negative and statistically negligible, while the economic growth lag is substantially favourable. Greenfield investment plays a positive role to spur economic growth. As developing countries lack capital, Greenfield investment contributes to improving the economic condition [60]. The analysis of this study confirmed that if there is a 1 unit increase in the Greenfield investment of the host country, then there would be a 0.07 unit increase in that country's economic growth. The result of this study is supported by the studies of [38,61]; the authors found Greenfield investment supportive for the economic condition of such countries. As Greenfield investment is considered as the blessing for economic growth, therefore such investment can create new jobs. Such investments can also upgrade the managerial skills and expertise through technological spillovers. Remittances also help improve the remittance-receiving families' lifestyle and thus can contribute to increasing economic growth by investing from their savings. Remittances were found to be statistically significant and positive in the economic development of host developing countries. The result of this study is supported by the researches of [62,63]. This study outcome shows ODA as negative to economic growth, and this negative impact may worsen the business climate in developing countries. The results of this study are in line with the study of [64]; the author suggested that government may adopt friendly policies which can effectively use aid in the business sector. Trade and inflation con-tribute statistically significant and negative, whereas the population role is positive but insignificant towards economic growth. Greenfield investment contributes to increasing the per capita income and improving the health status of the individual.

**Table 5:** Result of LA&C developing countries.

Variables	EG_*it*_	HEALTH_*it*_	EDU_*it*_	HDI_*it*_
Constant	−0.0577 (0.523)	−0.0097 (0.351)	−0.0121 (0.827)	−0.0402 (0.241)
Lag of each dependent variable	1.1172** (0.004)	0.9378*** (0.000)	0.9562***(0.000)	0.9781*** (0.000)
GFDI_*it*_	0.0699*** (0.000)	0.0059** (0.07)	0.0094** (0.011)	0.0099***(0.000)
REM_*it*_	0.0962*** (0.001)	0.0029** (0.031)	0.0065** (0.041)	0.0042** (0.042)
ODA_*it*_	−0.0069 (0.631)	−0.0004 (0.389)	−0.0074 (0.39)	−0.0066 (0.351)
TO_*it*_	−0.1481** (0.031)	0.0045 (0.338)	0.0339** (0.001)	−0.0425** (0.041)
POP_*it*_	0.0358 (0.103)	−0.0037** (0.039)	0.0058 (0.657)	−0.0121 (0.259)
INF_*it*_	−0.0001** (0.045)	−0.0022** (0.040)	−0.0024** (0.047)	−0.0004** (0.042)
Countries	23	23	23	23
Observations	418	418	418	418
AR(2)P-value	0.528	0.466	0.610	0.581
Sargen*/*hansen p-value	0.608	0.794	0.645	0.662

Brackets (.) denotes *P*-Value, *** and ** denotes 1 % and 5 % significance level.

Remittances in this study improve the health of the families who receive remittance, and the result of this study is in line with the outcome of [65]. On the other hand, ODA has an adverse health effect on the individual of devel-oping countries. These results are supported by the study of [66]. Greenfield investment is positive and statistically significant and contributes to the education sector of developing countries. The result is supported by Refs. [67,68]. Foreign investment helps in job creation that further leads to increase in enrollment rates and a more competent workforce are the results of greater pay, which motivate people to invest in their own education. ODA in this study is statistically insignificant and negative, whereas inflation is significantly negative related to education. Trade and population are also positive related, but the latter is statistically negligible. These results are in line with the study of [69]; the author found that ODA has no role while trade has a positive role in the education sector of developing countries. Aid is viewed as the worst tool for human development because it is ineffective and unable to improve conditions for people's health and education [70]. Aid in terms of Grants and concessional loan is often misused at gross root level. Though literature shows that through financing gap, trade gap, fiscal gap theories, aid helps in reducing poverty and increase per capita income.

Greenfield investment contributes to increasing income of household which further improve the education and health of individuals and the overall level of HDI of a host country. Greenfield investment is found as significantly positive, and the same result is seen by the study of [57,71]. The study concludes that Greenfield investment plays a very constructive role in bringing new technologies that will improve the skills of local labors. The administration of those firms can also arrange special training workshops for the improvement of existing staff performance. Remittances increase the income of the remittance-receiving families, which improve the life standard of individual, and this is also proven by Ref. [65]. The remittance earning families can best educate their child by providing educated environment and sends them to quality institutions for education. On the other hand, ODA harms the welfare of these developing countries because mostly aid is misused at the gross root level. Trade and inflation contribute statistically significant and negatively, whereas the population is insignificant and negative towards HDI.

The Variance Inflation Factor (VIF) is the panel data multicollinearity metric [72]. If VIF is less than 10, the results show no multicollinearity among variables [73]. This study result shows no multicollinearity among independent variables as each independent variable has a VIF value less than 10, as shown in Table 6. In conclusion, multicollinearity is identified using VIF in panel data analysis, which also helps with variable choice and model specification, increases the accuracy of parameter estimates, and ensures the validity of statistical inferences. In panel data analysis, researchers can improve the interpretability and reliability of their regression models by tackling multicollinearity.

**Table 6:** Multicollinearity test of LA&C developing countries.

Variables	VIF	1*/*VIF
GFDI_*it*_	3.28	0.304878
REM_*it*_	1.28	0.78125
ODA_*it*_	2.3	0.434298
TO_*it*_	1.67	0.598344
POP_*it*_	2.84	0.352113
INF_*it*_	1.54	0.64902

## 6 Conclusion and summary

In this research study, 23 LA&C developing countries for 1998–2017 are considered for the analysis. IPS and Sys- GMM methods are applied for stationarity testing and full results investigation. The results revealed that Greenfield investment is the most suitable investment that helps boost the economy, health, education and whole development of LA&C developing nations. Remittances have also contributed to economies by lifting individuals' income that further improved their siblings and children's health standard and education. On the other side, foreign aid is considered a curse, worsens the economy, and is not impressive enough to boost individual income, health, and education. From the policies management point of view, by making more welcoming business policies, emerging LA&C countries can draw more Greenfield investments. These nations should prioritise quality over quantity when it comes to Greenfield investment. To educate their labour with new technologies, companies should organize training and seminars for their employees. The state should also inform its citizens, which can help draw foreign investors. These nations are urged to install environmentally friendly and energy efficient technologies for production purposes in order to achieve this goal. Furthermore, rapid population expansion in these nations should be regulated by good management in order for inhabitants to benefit from improved environmental performance.

On the other hand, ODA harms developing LA&C countries’ health, education, economic development, and welfare. It is also advised to avoid ODA because it causes duct disease. Therefore, international aid should be avoided by LA&C governments to reduce their reliance on aid. The systems for monitoring and controlling aid are frequently insufficient in many developing countries. As a result, due to inadequate administration, there is a propensity for help to be mishandled at the grassroots level. Health activities in a large populated developed world are often limited by the population, as a large population negatively affects the health sector in developing countries. To manage the enormous birth population, the governments of developing LA&C countries should concentrate on health activities. It is imperative to increase trade volume and sign new trade agreements with developed countries since trade openness is universally acknowledged as a critical component of economic growth. LA&C countries may successfully raise the caliber and standards of their educational and medical institutions through boosting trade. The global problem of inflation is serious, especially for emerging countries where inflation rates are frequently high. Therefore, to manage and reduce inflation efficiently, nations in Latin America and the Caribbean should think about applying monetary or fiscal policy measures. This study is limited to a balanced panel data of LA&C nations and a future study could be conducted for middle-income and upper-middle income nations. A comprehensive future research could be conducted on the unbalanced LA&C countries and their results will be fruitful for the practitioners and policy makers.

## Data availability statement

Data included in article/supp. material/referenced in article.

## CRediT authorship contribution statement

**Ali Raza:** Conceptualization, Data curation, Formal analysis, Investigation, Methodology, Writing – original draft, Writing – review & editing. **Muhammad Imran Nadeem:** Conceptualization, Data curation, Formal analysis, Investigation, Methodology, Writing – original draft, Writing – review & editing. **Kanwal Ahmed:** Conceptualization, Data curation, Formal analysis, Investigation, Methodology, Writing – original draft, Writing – review & editing. **Izaz Hassan:** Conceptualization, Data curation, Formal analysis, Investigation, Methodology, Writing – original draft, Writing – review & editing. **Sayed M. Eldin:** Conceptualization, Data curation, Formal analysis, Investigation, Methodology, Writing – original draft, Writing – review & editing. **Nivin A. Ghamry:** Conceptualization, Data curation, Formal analysis, Investigation, Methodology, Writing – original draft, Writing – review & editing.

## Declaration of competing interest

The authors declare that they have no known competing financial interests or personal relationships that could have appeared to influence the work reported in this paper.

## References

[1] Soumare′ I. (2015). Does FDI improve economic development in North African countries. Appl. Econ..

[2] Raza A., Azam M., Tariq M. (2020). The impact of greenfield-FDI on socio-economic development of Pakistan. HSE Economic Journal.

[3] Phimmavong K. (2017). Impacts of foreign capital inflows on economic growth in 6 ASEAN countries: a panel data analysis. Int. J. Manag. Account. Econ..

[4] UNCTAD (2019).

[5] Unctad S. (2020). https://bit.ly/21GbfKX.

[6] Mohamed S.E., Sidiropoulos M.G. (2010). Another look at the Determinants of foreign direct investment in MENA countries: an empirical investigation. J. Econ. Dev..

[7] Bayar Y. (2017). Greenfield and Brownfield investments and economic growth: evidence from central and eastern European union coun- tries. Naše gospodarstvo/Our economy.

[8] Stepanok I. (2015). Cross-border mergers and greenfield foreign direct investment. Rev. Int. Econ..

[9] Meyer K.E. (2003).

[10] Wang M., Sunny-Wong M.C. (2009). What drives economic growth. The case of cross-border M&A and greenfield FDI activities. Kyklos.

[11] Raza A., Tariq M., Sadiqa B.A. (2021). An Empirical data investigation of the Greenfield investment: welfare nexus from low-income countries. International Journal of Innovation, Creativity and Change.

[12] Qiu L.D., Wang S. (2011). FDI policy, greenfield investment and cross‐border mergers. Rev. Int. Econ..

[13] Abu-Bakar N.A., Raji J.O., Adeel-Farooq R.M. (2019). Greenfield, mergers and acquisitions, energy consumption and environmental performance in selected SAARC and ASEAN countries. Int. J. Energy Econ. Pol..

[14] Norbäck P.J., Persson L. (2004). Privatization and foreign competition. J. Int. Econ..

[15] Raza A., Azam M.K., Tariq M. (2020). A panel data investigation of greenfield investment on the welfare of african developing countries. Int. Rev. Soc. Sci..

[16] World Bank (2020).

[17] Caldero′n C., Loayza N., Serve′n L. (2004).

[18] Solow R.M. (1956). A contribution to the theory of economic growth. Q. J. Econ..

[19] Swan T.W. (1956). Economic growth and capital accumulation. Econ. Rec..

[20] De Mello L.R. (1997). Foreign direct investment in developing countries and growth: a selective survey. J. Dev. Stud..

[21] Romer P. (1993). Idea gaps and object gaps in economic development. J. Monetary Econ..

[22] Moon H.C., Kim H.K., Lee D.H. (2003).

[23] Neto P., Brandão A., Cerqueira A. (2008). The impact of FDI, cross border mergers and acquisitions and greenfield investments on economic growth. The IUP Journal of Business Strategy.

[24] Slangen A.H., Hennart J.F. (2008). Do multinationals really prefer to enter culturally distant countries through Greenfields rather than through acquisitions. The role of parent experience and subsidiary autonomy. J. Int. Bus. Stud..

[25] Almsafir A.M.K., Latif N.W.A., Bekhet H.A. (2011). Analyzing the green field investment in Malaysia from 1970 to 2009: a bound testing approach. Australian Journal of Basic and Applied Sciences.

[26] Park C.Y., Byun H.S., Lee H.H. (2012). Assessing factors affecting M&as versus greenfield FDI in emerging countries. Papers and briefs. Economics Working Papers.

[27] Zhuang H., Griffith D. (2013). The effect of mergers & acquisitions and greenfield FDI on income inequality. Int. J. Appl. Econ..

[28] Harms P., Méon P.G. (2013). November). The growth effects of greenfield investment and mergers and acquisitions: econometric investigation and implication for MENA countries.

[29] Harms P., Me′on P.G. (2013). Economic Research Forum Working Paper.

[30] Ashraf A., Herzer D. (2014). The effects of greenfield investment and M&As on domestic investment in developing countries. Appl. Econ. Lett..

[31] Eren M., Zhuang H. (2015). Mergers and acquisitions versus greenfield investment, absorptive capacity, and economic growth: evidence from 12 new member states of the European Union. E. Eur. Econ..

[32] Luu H. (2016). Greenfield investments, cross-border M&A's, and economic growth in emerging countries. Economic and Business Letter.

[33] Marinescu N. (2016). Greenfields and acquisitions: a comparative analysis. Bulletin of the Transilvania University of Brasov. Economic Sciences. Series V.

[34] Ashraf A., Herzer D., Nunnenkamp P. (2016). The effects of Greenfield FDI and cross-border M&A's on total factor productivity. World Econ..

[35] Amoroso S., Castello P.M. (2018). Inward greenfield FDI and patterns of job polarization. Sustainability.

[36] Amoroso S., Müller B. (2018). The short-run effects of knowledge intensive greenfield FDI on new domestic entry. J. Technol. Tran..

[37] Owen E. (2019). Foreign direct investment and elections: the impact of greenfield FDI on incumbent party reelection in Brazil. Comp. Polit. Stud..

[38] Luu H.N., Nguyen N.M., Ho H.H., Nam V.H. (2019). The effect of corruption on FDI and its modes of entry. Journal of Financial Economic Policy.

[39] Zhang Z., Zhang G., Su B. (2022). The spatial impacts of air pollution and socio-economic status on public health: empirical evidence from China. Soc. Econ. Plann. Sci..

[40] Zhang Z., Zhang G., Li L. (2022). The spatial impact of atmospheric environmental policy on public health based on the mediation effect of air pollution in China. Environ. Sci. Pollut. Control Ser..

[41] Raza A., Akbar S., Sadiqa B.A. (2021). Relationship among economic growth, health, education, economic development and green- field investment as mode of FDI: evidence from MENA countries. Int. Rev. Soc. Sci..

[42] Wdi T. (2020).

[43] UNDP H.D.R. (2020). http://hdr.undp.org/en/content/2020-human-development-index-ranking.

[44] Lehnert K., Benmamoun M., Zhao H. (2013). FDI inflow and human development: analysis of FDI's impact on host countries' social welfare and infrastructure. Thunderbird Int. Bus. Rev..

[45] Barro R.J. (1991). Economic growth in a cross section of countries. Q. J. Econ..

[46] Romer P.M. (1986). Increasing returns and long-run growth. J. Polit. Econ..

[47] UNDP U. (1990).

[48] Kosack S., Tobin J. (2006).

[49] Raza A., Iqbal M., Hussain N. (2021). Is Greenfield investment greener for the welfare of lower-middle income countries? Market-based empirical analysis with GMM approach. Journal of Marketing Strategies.

[50] Wooldridge J.M. (2006).

[51] Arellano M., Bond S. (1991). Some tests of specification for panel data: Monte Carlo evidence and an application to employment equations. Rev. Econ. Stud..

[52] Arellano M., Bover O. (1995). Another look at the instrumental variable estimation of error-components models. J. Econom..

[53] Blundell R., Bond S. (1998). Initial conditions and moment restrictions in dynamic panel data models. J. Econom..

[54] Roodman D. (2009). How to do xtabond2: an introduction to difference and system GMM in Stata. STATA J..

[55] Aggarwal R., Demirgüc-Kunt A., Peria M.S.M. (2010). Do remittances promote financial development. J. Dev. Econ..

[56] Sharma B., Gani A. (2004). The effects of foreign direct investment on human development. Global Economy Journal.

[57] Tintin C. (2012).

[58] Pesaran M.H. (2007). A simple panel unit root test in the presence of cross-section dependence. J. Appl. Econom..

[59] Levin A., Lin C.F., Chu C.S.J. (2002). Unit root tests in panel data: asymptotic and finite-sample properties. J. Econom..

[60] Raza A., Akbar S., Raza Z. (2021). Does Greenfield-foreign direct investment inflow contribute in socioeconomic develop- ment? Empirical evidence from developing countries. Pakistan Journal of Humanities and social sciences.

[61] El′ıas S., Fernandez M.D.R. (2000). Human capital investment, income levels and economic growth in Latin American countries. Paper presented at 40th Congress of the European Regional Science Association, Barcelona, Spain.

[62] Burger M.J., Ianchovichina E.I. (2017). Surges and stops in greenfield and M&A FDI flows to developing countries: analysis by mode of entry. Rev. World Econ..

[63] Ferraz D., Moralles H.F., Campoli J.S., Oliveira F.C.R.D., Rebelatto D.A.D.N. (2018). Economic complexity and human develop- ment: DEA performance measurement in Asia and Latin America. Gestão & Produção.

[64] Adedokum A.J. (2017). Foreign aid, governance and economic growth in Sub-Saharan Africa: does one cap fit all. Afr. Dev. Rev..

[65] Williamson C.R. (2008). Foreign aid and human development: the impact of foreign aid to the health sector. South. Econ. J..

[66] Lopez J.H., Fajnzylber P., Acosta P. (2007).

[67] Burns D.K., Jones A.P., Goryakin Y., Suhrcke M. (2017). Is foreign direct investment good for health in low and middle income countries. An instrumental variable approach. Soc. Sci. Med..

[68] Kim Y.H. (2009). Cross-border M&A verses greenfield FDI: economic integration and its welfare impact. J. Pol. Model..

[69] Trevino L.J., Daniels J.D., Arbelaez H. (2002). Market reform and FDI in Latin America: an empirical investigation. Transnatl. Corp..

[70] Stojanov R., Strielkowski W. (2013). The role of remittances as more efficient tool of development aid in developing countries. Prague Econ. Pap..

[71] Alexandersson L., Myrbäck G. (2017).

[72] Salmero′n-Go′mez R., Garc′ıa-Pe′rez J., Lo′pez-Mart′ın M.D.M., Garc′ıa C.G. (2016). Collinearity diagnostic applied in ridge estimation through the variance inflation factor. J. Appl. Stat..

[73] Neter J., Wasserman W., Kutner M.H. (1985).

